# Smartphone-Based versus Non-Invasive Automatic Oscillometric Brachial Cuff Blood Pressure Measurements: A Prospective Method Comparison Volunteer Study

**DOI:** 10.3390/jpm14010015

**Published:** 2023-12-21

**Authors:** Lila Delmotte, Olivier Desebbe, Brenton Alexander, Karim Kouz, Sean Coeckelenbergh, Patrick Schoettker, Tuna Turgay, Alexandre Joosten

**Affiliations:** 1 Department of Anesthesiology, Erasme University Hospital, Université Libre de Bruxelles, 808 Route de Lennik, 1070 Brussels, Belgium; lila.delmotte@ulb.be (L.D.); turgay.tuna@hubruxelles.be (T.T.); 2 Department of Anesthesiology & Perioperative Medicine, Sauvegarde Clinic, Ramsay Santé, 69009 Lyon, France; oldesebbe@yahoo.com; 3 Department of Anesthesiology, University of California San Diego, La Jolla, CA 92103, USA; brentonetone@gmail.com; 4 Department of Anesthesiology, Center of Anesthesiology and Intensive Care Medicine, University Medical Center Hamburg-Eppendorf, Martinistrasse 52, 20246 Hamburg, Germany; 5 Department of Anesthesiology, Université Paris-Saclay, Paul Brousse Hospital, Assistance Publique Hôpitaux de Paris (APHP), 94800 Villejuif, France; 6 Outcomes Research Consortium, Cleveland, OH 44195, USA; 7 Biospectal SA, 1003 Lausanne, Switzerland; patrieck.schoettker@chuv.ch; 8 Department of Anesthesiology, Lausanne University Hospital, University of Lausanne, 1011 Lausanne, Switzerland; 9 Department of Anesthesiology and Perioperative Medicine, David Geffen School of Medicine, University of California Los Angeles, Los Angeles, CA 90095, USA

**Keywords:** arterial hypertension, mobile phone, mobile health, digital health, accuracy, precision, blood pressure, arterial pressure

## Abstract

**Introduction:** Mobile health diagnostics have demonstrated effectiveness in detecting and managing chronic diseases. This method comparison study aims to assess the accuracy and precision of the previously evaluated OptiBP™ technology over a four-week study period. This device uses optical signals recorded by placing a patient’s fingertip on a smartphone’s camera to estimate blood pressure (BP). **Methods:** In adult participants without cardiac arrhythmias and minimal interarm blood pressure difference (systolic arterial pressure (SAP) < 15 mmHg or diastolic arterial pressure (DAP) < 10 mmHg), three pairs of 30 s BP measurements with the OptiBP™ (test method) were simultaneously compared using three pairs of measurements with the non-invasive oscillometric brachial cuff (reference method) on the opposite arm over a period of four consecutive weeks at a rate of two measurements per week (one in the morning and one in the afternoon). The agreement of BP values between the two technologies was analyzed using Bland–Altman and error grid analyses. The performance of the smartphone application was investigated using the International Organization for Standardization (ISO) definitions, which require the bias ± standard deviation (SD) between two technologies to be lower than 5 ± 8 mmHg. **Results:** Among the 65 eligible volunteers, 53 participants had adequate OptiBP™ BP values. In 12 patients, no OptiBP™ BP could be measured due to inadequate signals. Only nine participants had known chronic arterial hypertension and 76% of those patients were treated. The mean bias ± SD between both technologies was −1.4 mmHg ± 10.1 mmHg for systolic arterial pressure (SAP), 0.2 mmHg ± 6.5 mmHg for diastolic arterial pressure (DAP) and −0.5 mmHg ± 6.9 mmHg for mean arterial pressure (MAP). Error grid analyses indicated that 100% of the pairs of BP measurements were located in zones A (no risk) and B (low risk). **Conclusions:** In a cohort of volunteers, we observed an acceptable agreement between BP values obtained with the OptiBP^TM^ and those obtained with the reference method over a four-week period. The OptiBP^TM^ fulfills the ISO standards for MAP and DAP (but not SAP). The error grid analyses showed that 100% measurements were located in risk zones A and B. Despite the need for some technological improvements, this application may become an important tool to measure BP in the future.

## 1. Introduction

The increasing prevalence of arterial hypertension in patients throughout the world, regardless of socioeconomic status, continues to highlight this disease as a major public health problem [1,2,3]. It is essential to detect arterial hypertension at an early stage and consistently monitor blood pressure (BP) to prevent its complications. To definitively diagnose chronic hypertension, the European Society of Hypertension recommends repeated BP measurements using either the auscultatory or non-invasive automatic oscillometric method, either in a doctor’s office or at an out-of-hospital monitoring facility [4,5]. It is worth noting that out-of-hospital monitoring prevents white-coat syndrome [6,7,8].

The last two decades have seen the emergence of numerous mobile phone applications [9,10]. Some applications can now measure vital signs, provide advanced hemodynamic variables, such as cardiac index and/or pulse pressure variation [11,12,13,14], or even measure cardiac conditions, such as ejection fraction [14]. The use of a smartphone application to measure BP on a daily basis has the potential to greatly improve the management of arterial hypertension. This is particularly true in low-income countries, where the availability of smartphones is high while access to health care is low [15,16,17].

When it comes to BP measurements, auscultatory and automated oscillometric sphygmomanometers are the standard of care reference techniques [18,19,20]. Cuffless approaches using smartphone-based medical applications have recently emerged as promising new technologies to detect and monitor hypertension [21,22,23]. A smartphone, for example, is a very promising tool as it is accessible to a wide range of the population in both industrialized and developing countries [24].

Recently, a Swiss MedTech start-up (Biospectal, Lausanne, Switzerland) developed a smartphone application named “Optical Blood Pressure” or OptiBP™ to measure BP using the principle of photoplethysmography [25,26,27,28,29,30,31,32]. This non-invasive method relies on the detection of a pulsed light wave captured by placing the user’s finger on the smartphone camera to retrieve transdermal optical signals from the fingertips. An algorithm estimates BP values from the pulse wave morphology. This is similar to pulse oximetry, which is used to measure oxygen saturation [33,34]. This innovative method has the potential to significantly improve global accessibility of early detection and monitoring for hypertension. This approach also encourages the active participation of patients in their own health [35].

Several studies, mainly conducted in perioperative settings (emergency department, post-anesthesia care unit, and intensive care unit), have compared BP values obtained using the OptiBP™ system with those obtained using a brachial cuff or an arterial line [26,27,28,29,30,31]. All of these studies reported an acceptable agreement between the OptiBP™ system and the reference method during relatively short study periods (from a few minutes to a maximum of two consecutive days).

This method comparison study aimed to assess the accuracy and precision of BP values collected using OptiBP™ (tested method) against those obtained with a non-invasive automatic oscillometric brachial cuff (reference method) over a period of four consecutive weeks. This approach thus assessed OptiBP™’s long-term performance using longitudinal data on a cohort of volunteers.

## 2. Materials and Methods

This study was approved by the Erasme University Hospital Ethics Committee in Brussels, Belgium on 8 February 2023, under reference P2022/410. All participants provided written informed consent before the beginning of the study.

All adult participants > 18 years of age who were available for four consecutive weeks were eligible except for those with known cardiac arrhythmias or BP differences (systolic arterial pressure (SAP) > 15 mmHg or diastolic arterial pressure (DAP) > 10 mmHg) between arms.

### 2.1. Test Method (OptiBP^TM^)

The OptiBP™ version 4.09 was installed on a Google Pixel 5^®^ (Mountain View, CA, USA) equipped with the clinical version of OptiBP™, named CamBP™. This version uses the same functionality as OptiBP™ and the same algorithm to convert optical signals into BP values. To prevent investigator and participant bias, no BP values were displayed on the smartphone screen when the investigator used the smartphone. The results were stored directly onto a secure cloud storage system to which only the engineers in charge of the analyses had access. Light emitted from the Google Pixel 5^®^ was passed through each patient’s fingertip, reflecting differently off the tissues as the volume of blood flow changed there. The light passing back to the smartphone camera’s image sensor generated images that were processed using the OptiBP application to calculate the BP. The methodology of optical signal acquisition and its initial processing have been published elsewhere recently.

### 2.2. Reference Method (Non-Invasive Automatic Brachial Cuff Oscillometry)

The automatic brachial cuff used in this study was an Omron X3 Comfort (Kyoto, Japan), validated for professional use in accordance with the standards set by the European Society of Hypertension. Two sizes of cuff were supplied: M size (22–32 cm) and L size (32–42 cm).

Each participant was assigned a sequentially coded booklet (001, 002, etc.) to ensure confidentiality. The booklet contained demographic information (age, biological sex, height, weight, hypertension status, and current treatments) and a list of inclusion and exclusion criteria. BP measurements obtained during the various visits were recorded and, where appropriate, annotations relating to problems using the OptiBP™ (system malfunctions, etc.). Additionally, an electronic backup was made in an individualized table using Microsoft Excel^®^ (Redmond, WA, USA) software, version 2019.

### 2.3. Study Protocol

This study was carried out over eight measurement sessions during four consecutive weeks, twice a week (one in the morning and one in the afternoon).

The aim of the first visit was to check for eligibility, obtain informed consent from the participants, collect demographic data, prepare the subject for taking BP measurements, provide training on the use of OptiBP™, and perform calibration of the OptiBP™. Calibration is an essential step and was performed once during the first visit in order to establish a BP reference and absolute values of the individual’s BP. Two pairs of three 30 s measurements collected using OptiBP™ were simultaneously compared to two pairs of BP measurements collected using the reference method. Following this initial calibration, participants had seven follow-up sessions. Three pairs of three 30 s measurements with the OptiBP™ were simultaneously compared to three pairs of BP measurements collected using the reference method. Patients were asked to empty their bladder if needed preceding measurements. Participants were then instructed to remain in a seated position for at least five minutes, with their back, elbows, and forearms supported; legs uncrossed; and feet flat on the floor. An appropriately sized cuff was attached to the participant’s arm, and the arms were positioned on a table at the level of the heart. Systematically, BP measurements were taken on the left arm using an automatic brachial cuff, while the OptiBP™ was used on the index or middle finger of the right hand. The fingertip was positioned and kept motionless on the smartphone camera with balanced force. When activated via the OptiBP™ software, a 30 s high-speed video recording was made using the lamp next to the camera in order to capture the volumetric variations in blood flow through the finger pulp. The optical pulse wave captured was then analyzed using the above-described photoplethysmography to estimate BP. The software provided visual feedback in the form of a green image if the measurement was correct and as an orange image if the finger was incorrectly positioned, causing excessive light to enter between the finger and the camera.

### 2.4. Statistical Analysis

No sample size was calculated for this study. As it was a pilot study, we decided to include at least 50 participants. Patient characteristics are presented as mean ± standard deviation (SD) or absolute number and percentage (%). SAP, diastolic arterial pressure (DAP), and mean arterial pressure (MAP) values obtained with the OptiBP^TM^ were compared with those obtained using the reference method using Bland–Altman analyses by calculating the mean bias (BP of the test method minus BP of the reference method) together with SD, and 95% limits of agreement (mean of the difference ± 1.96 × SD) accounting for repeated measurements. We assessed the performance of the OptiBP^TM^ with the International Organization for Standardization (ISO) ISO81060-2:2018 standards [36], which require the bias ± SD between the test and the reference method to be ≤5.0 mmHg ± 8.0 mmHg.

An error grid analysis method that has been recently proposed by Saugel et al. was also performed [37,38]. This analysis consists of a scatterplot with reference BP measurements on the *x*-axis and measurements from the test method on the *y*-axis overlaid on a grid that is divided into five risk zones (zones A to E). Each BP measurement pair was categorized into one of the five risk zones, which describes the potential clinical risk caused by a difference in the BP measured using the test method and that measured using the reference method. These five zones are color-coded from green (zone A, no risk) to red (zone E, life-threatening risk). This method to assess BP method comparison studies is now widely used as shown in recent publications [39,40,41,42]. All statistics were performed using Microsoft Excel^®^ (Redmond, WA, USA) and MedCalc^®^ Statistical Software version 19.6.4 (MedCalc Software Ltd., Ostend, Belgium), and the error grid analysis was performed using MATLAB^®^ (The MathWorks Inc., Natick, MA, USA).

## 3. Results

**Figure 1:**
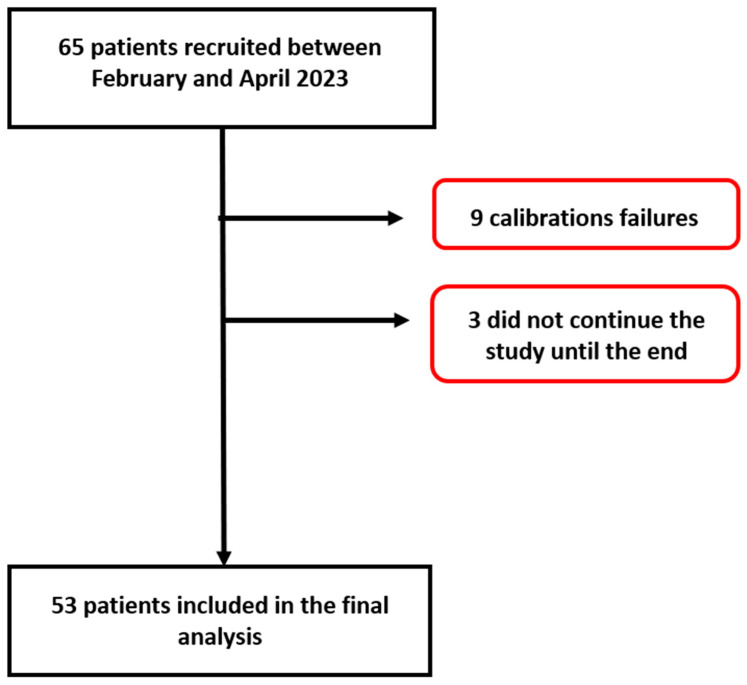
Patient flow chart.

Among the 65 participants recruited between 8 February 2023 and 2 April 2023, 53 participants were included in the statistical analyses. Nine participants (15% of study collective) were excluded because the initial calibration of the OptiBP™ failed, and three participants dropped out of the study (5%). Reasons for calibration failure were diverse but included artefacts due to hand movements and physiological disturbances to blood flow capture (peripheral vascular disease or low hand temperature). A flow chart is shown in Figure 1.

Of the 53 studied volunteers, 51% were younger than 50 years and 70% were women. Only 17% of the participants had arterial hypertension, and 76% of that group were treated pharmacologically. The body max index was 26 ± 5 kg/m^2^, and 60% of the participants were from Europe (Table 1).

**Figure 2:**
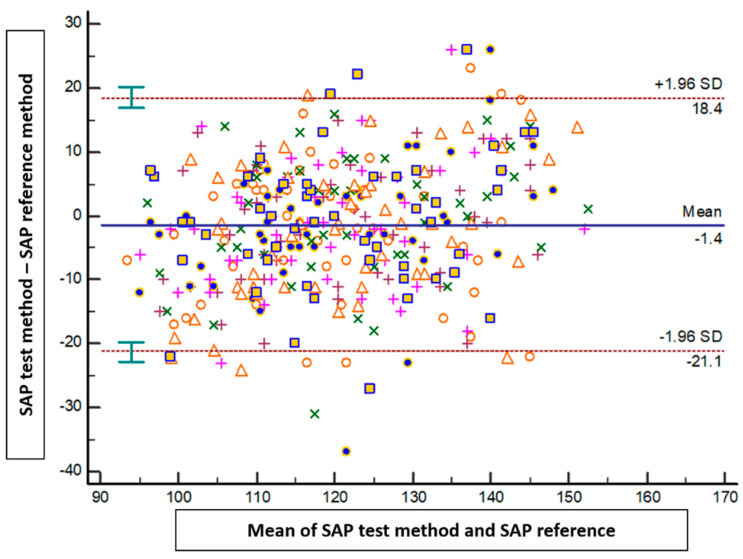
Blant-Altman analysis of systolic arterial pressure measured either at the arm or using the smartphone application. Each point represents measurement pairs. SAP: systolic arterial pressure.

**Figure 3:**
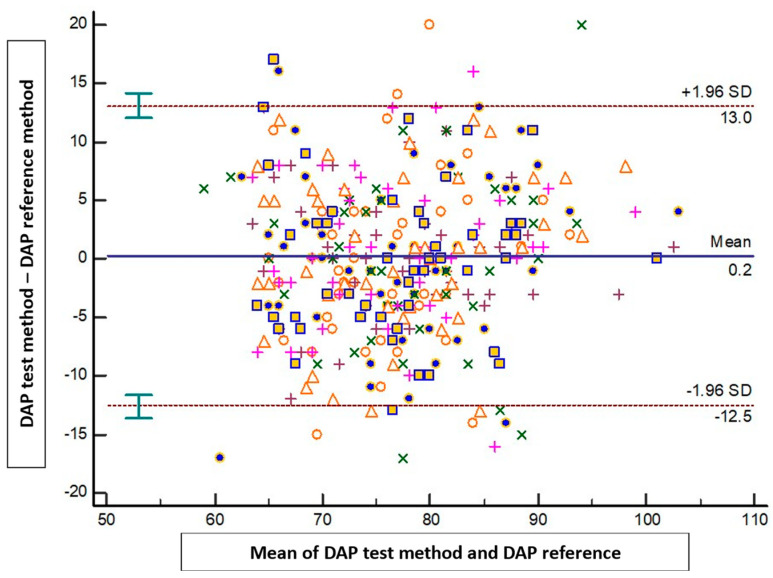
Blant-Altman analysis of diastolic arterial pressure measured either at the arm or using the smartphone application. Each point represents measurement pairs. DAP: diastolic arterial pressure.

**Figure 4:**
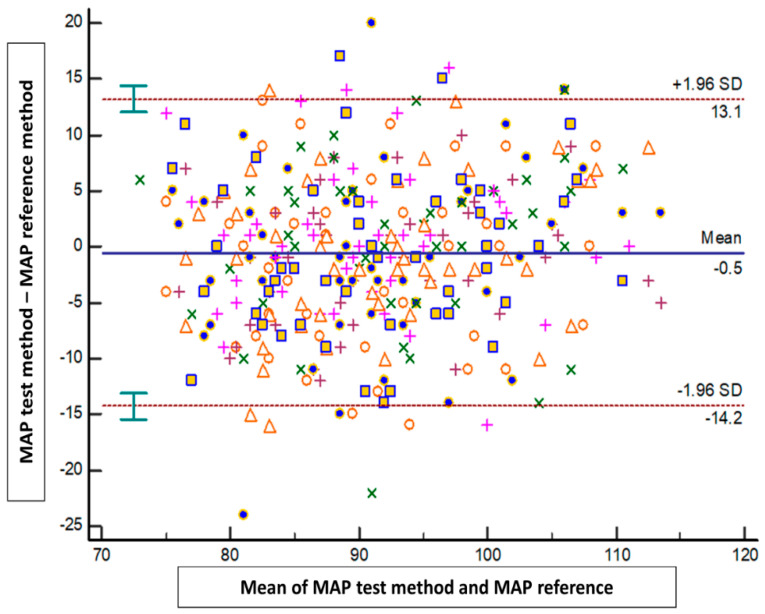
Blant-Altman analysis of mean arterial pressure measured either at the arm or using the smartphone application. Each point represents measurement pairs. MAP: mean arterial pressure.

The results of the Bland–Altman analysis revealed a mean bias ± standard deviation (limits of agreement) between the tested method and the reference method of −1.4 mmHg ± 10.1 mmHg (−21.1 to 18.4 mmHg) for SAP, 0.2 mmHg ± 6.5 mmHg (−12.5 to 13 mmHg) for DAP, and −0.5 mmHg ± 6.9 mmHg (−14.2 to 13.1 mmHg) for MAP (Figure 2, Figure 3 and Figure 4).

**Figure 5:**
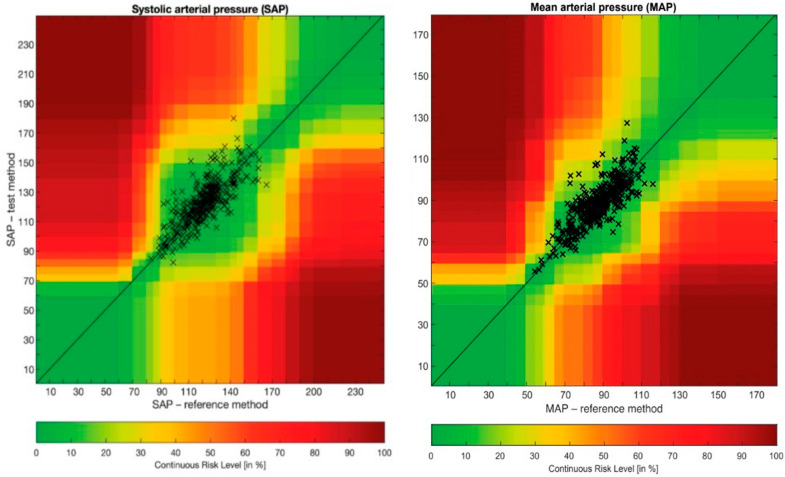
Error grid analysis of systolic arterial pressure and mean arterial pressure. MAP: mean arterial pressure; SAP: systolic arterial pressure.

Error grid analysis revealed that the proportions of pairs of SAP and MAP measurements were 96.2% in zone A and 3.8% in zone B, and 94.7% in zone A and 5.3% in zone B, respectively (Figure 5).

Error grid analysis was also performed at each time point and revealed similar accuracy and precision. All pairs of measurements were situated in risk zones A and B, and values ranged from 94 to 98% for SAP and from 90 to 100% for MAP. At time point 1, the proportions of pairs of SAP and MAP measurements were 94% in zone A and 6% in zone B, and 96% in zone A and 4% in zone B, respectively. At time point 2, the proportions of pairs of SAP and MAP measurements were 98% in zone A and 2% in zone B, and 100% in zone A and 0% in zone B, respectively. At time point 3, the proportions of pairs of SAP and MAP measurements were 98% in zone A and 2% in zone B, and 96% in zone A and 4% in zone B, respectively. At time point 4, the proportions of pairs of SAP and MAP measurements were 96% in zone A and 4% in zone B, and 96% in zone A and 4% in zone B, respectively. At time point 5, the proportions of pairs of SAP and MAP measurements were 96% in zone A and 4% in zone B, and 90% in zone A and 10% in zone B, respectively. At time point 6, the proportions of pairs of SAP and MAP measurements were 98% in zone A and 2% in zone B, and 96% in zone A and 4% in zone B, respectively. At time point 7, the proportions of pairs of SAP and MAP measurements were 94% in zone A and 6% in zone B, and 92% in zone A and 8% in zone B, respectively.

## 4. Discussion

In this prospective pilot method comparison study conducted on volunteers over a four-week study period, we observed an acceptable agreement between a novel blood pressure application (OptiBP™) and the reference method. MAP and DAP reached the ISO81060-2:2018 standards, but SAP did not. However, 100% of the measurement pairs were located in the error grid risk zones A and B (no- or low-risk zones). The results of the present study are in agreement with previous studies conducted in Europe and Africa/Asia. However, in all previous studies, the study period was very short, ranging from a few minutes to maximum of two consecutive days. As such, this study is the first to provide longitudinal data on this new technology.

Currently, there are only seven studies that have compared BP measurements collected with the OptiBP™ versus a non-invasive or invasive reference method. Three studies were carried out in Lausanne, Switzerland, by the inventors of the BP application [26,28,31], three other studies by our team in Belgium [27,29,30] and the last study was conducted as a tri-center study in South Africa, Tanzania, and Bangladesh [43]. These studies are summarized in Table 2. The studies carried out in Switzerland exhibited the best results, perhaps due to a higher quality of BP measurements. Two of the studies had compared OptiBP™ to the auscultatory method on almost a hundred patients and revealed a good concordance of BP measurements between both technologies [26,31]. All BP measurements were within ISO81060-2:2018 standards. However, this study was carried out in a very controlled study environment of patients followed in a hypertension clinic. Another recently published study was conducted on a cohort of 119 patients equipped with a radial arterial catheter who underwent non-cardiac surgery [28]. The study took place during the induction of general anesthesia when BP decreased due to vasoplegia and then transiently increased during intubation. A more recent study included obstetric women in Africa and Asia. The results demonstrated that BP measurements also fell within the ISO81060-2:2018 standards [43]. It should be noted that the studies described above did not report error grid analysis, with the exception of the study carried out during the induction of general anesthesia (the study contained a small amount of recorded BP in zone C, which indicated moderate risk for the patient). The studies conducted in Belgium had similar results to those of the present study. Several reports have thus shown the potential application of smartphones in measuring BP. However, this kind of technology is not perfect, as we observed in our study, with a 15% failure rate during calibration. This indicates that in a subset of patients (to be better defined in the future), OptiBP^TM^ cannot detect blood volume and display any BP value.

The main strength of this study was the repeated measurements of BP at different times of the day over a prolonged period (four consecutive weeks), thus coming closer to a realistic environment that simulates real-life conditions and clinically relevant BP monitoring. In addition, this study was conducted by a single non-invasive blood pressure monitor, eliminating intermonitor variations in BP measurements.

This study also has limitations that need to be highlighted. Firstly, the sample size was relatively small (53 participants), and the study population was mostly female (70%). However, we may consider that 53 participants were adequate for a pilot study and allows for the ability to calculate an adequate sample size for a larger future study. Secondly, only 17% of participants had chronic hypertension, a relatively small percentage for a study evaluating a technology intended to be used by hypertensive patients around the world. Future studies should be designed to include older patients and a higher percentage of hypertensive patients. It is also worth mentioning again the limited, but consistent, percentage of patients in which the technology could not appropriately calibrate. This will need to be examined further in future studies to determine if there is a consistent characteristic among these patients that may allow for improvements or adjustments.

### Future Prospects

Mobile phones are powerful communication devices, first demonstrated by Motorola in 1973, and made commercially available since 1984. In the last few years, mobile phones have become an integral part of our lives. The number of mobile cellular subscriptions is constantly increasing every year. In 2016, there were more than seven billion users worldwide. The percentage of internet usage also increased seven-fold globally from 6.5% to 43% between 2000 and 2020. Today, almost all of us have a smartphone in our pocket. However, we do not often use it to directly monitor or improve our health. This may change in the future, given the increasing number of physiological variables we can measure using a smartphone, with or without connected sensors [44,45,46,47,48]. Many applications are already “CE”-marked or FDA-cleared for medical use. However, validation and utility studies remain rare and are required before widespread clinical adoption. Data protection and privacy will also be important issues that need to be addressed before encouraging patients and clinicians to use these new smartphone applications [49,50,51].

## 5. Conclusions

In a cohort of predominantly female volunteers with overwhelmingly normal BP values, we observed an acceptable agreement between BP values obtained using the OptiBP^TM^ and BP values obtained with the widely used reference method. OptiBP^TM^ values fulfilled the requirements of the ISO for MAP and DAP. However, it is important to note that in 15% of participants, no BP values could be measured. All results should be considered preliminary data, and they will require further studies with a larger sample for external validation. Multiple ongoing studies will enable the acquisition of more data, and continuous improvements in data analysis will result from the constant evolution of artificial intelligence and machine learning. Every step in the algorithm can therefore be improved. In this regard, future improvements in the OptiBP^TM^ software should focus on increasing precision of higher measured values and the inclusion of a higher percentage of hypertensive patients. The future seems bright as using smartphones as tools to follow BP makes clinical follow-up accessible to a large part of the population that owns a smartphone at every layer of society, including in low- and middle-income countries.

## Figures and Tables

**Table 1:** Basic characteristics of the participants.

Variables	N = 53
Age (years)	47 ± 16
Sex, Male (N, %)	16 (30%)
Height (cm)	169 ± 9
Body weight (kg)	73 ± 14
Body mass index (kg/m^2^)	26 ± 5
Ethnicity (N, %) European North African Asian African	32 (60%) 8 (15%) 10 (19%) 3 (6%)
Chronic hypertension (N, %)	9 (17%)
Untreated hypertensive patients (N, %)	2 (4%)
Hypertensive patients with a treatment (N, %)	7 (13%)

**Table 2:** Summary of the various studies conducted on the OptiBP™.

Author	Year	Place of Investigation and Reference Method	Population	No. of Subjects	Duration of the Study	ISO Standards	Error Grid
Schoettker et al. [32]	2020	Hypertension clinic and auscultatory method	Hypertensive or non-hypertensive patients	40	7 × 1 min	SAP: −0.7 ± 7.7 mmHg DAP: −0.4 ± 4.5 mmHg MAP: −0.6 ± 5.2 mmHg	Not realized
Degott et al. [31]	2021	Hypertension clinic and auscultatory method	Patients with hypertension	91	9 × 1 min	SAP: 0.5 ± 7.7 mmHg DAP: 0.4 ± 4.6 mmHg	Not realized
Desebbe et al. [30]	2022	Emergency department and automatic brachial cuff	General population	110	3 × 1 min	SAP: −0.1 ± 11.5 mmHg DAP: −0.1 ± 6.5 mmHg MAP: −0.3 ± 8.9 mmHg	SAP: A: 89.3, B: 10.7, C-E: 0 MAP: A: 86.9, B: 13.1, C-E: 0
Desebbe et al. [29]	2022	Recovery room and automatic brachial cuff	Post abdominal surgery	120 (101)	Each 15 min for 2 consecutive hours	SAP: 1.95 ± 11.0 mmHg DAP: 1.27 ± 8.0 mmHg MAP: 1.3 ± 7.0 mmHg	SAP: A: 89.9, B: 9.1, C: 1.0, D-E: 0 MAP: A: 90.3, B: 9.7, C-E: 0
Desebbe et al. [27]	2022	ICU and radial arterial catheter	Intensive care patients	22	Each hour for 5 consecutive hours during 2 consecutive days	SAP: 0.2 ± 13.75 mmHg DAP: 1.1 ± 5.97 mmHg MAP: 0.9 ± 7.27 mmHg	SAP: A: 88.4, B: 8.6, C: 3.0, D-E: 0 MAP: A: 88.8, B: 10.0, C: 1.0, D-E: 0
Hofmann et al. [28]	2023	Operating theatre and arterial catheter	Elective surgery	119	10 × 1 min	SAP: 0.0 ± 7.5 mmHg DAP: 0.1 ± 2.9 mmHg MAP: 0.1 ± 4.2 mmHg	SAP: A: 89.8, B: 9.0, C: 1.2, D-E: 0 MAP: A: 89.9, B: 9.8, C: 0.2, D-E: 0
Festo et al. [43]	2023	General population and auscultatory method	General and pregnant population	100 60	4 × 30 s	In South Africa SAP: 0.5 ± 5.8 mm Hg DAP: 0.1 ± 3.9 mmHg In Tanzania SAP: 0.8 ± 7.0 mmHg DAP: −4.0 ± 4.0 mmHg In Bangladesh SAP: 3.3 ± 7.4 mmHg DAP: −0.4 ± 4.3 mmHg	Not realized

## Data Availability

The data presented in this study are available on request from the corresponding author.

## References

[1] Mills K.T., Stefanescu A., He J. (2020). The global epidemiology of hypertension. Nat. Rev. Nephrol..

[2] NCD Risk Factor Collaboration (NCD-RisC) (2021). Worldwide trends in hypertension prevalence and progress in treatment and control from 1990 to 2019: A pooled analysis of 1201 population-representative studies with 104 million participants. Lancet.

[3] Boateng E.B., Ampofo A.G. (2023). A glimpse into the future: Modelling global prevalence of hypertension. BMC Public Health.

[4] Williams B., Mancia G., Spiering W., Agabiti Rosei E., Azizi M., Burnier M., Clement D.L., Coca A., de Simone G., Dominiczak A. (2018). 2018 ESC/ESH Guidelines for the management of arterial hypertension: The Task Force for the management of arterial hypertension of the European Society of Cardiology and the European Society of Hypertension: The Task Force for the management of arterial hypertension of the European Society of Cardiology and the European Society of Hypertension. J. Hypertens..

[5] Schutte A.E., Kollias A., Stergiou G.S. (2022). Blood pressure and its variability: Classic and novel measurement techniques. Nat. Rev. Cardiol..

[6] Pioli M.R., Ritter A.M., de Faria A.P., Modolo R. (2018). White coat syndrome and its variations: Differences and clinical impact. Integr. Blood Press. Control.

[7] Reynolds K., Bowling C.B., Sim J.J., Sridharan L., Harrison T.N., Shimbo D. (2015). The Utility of Ambulatory Blood Pressure Monitoring for Diagnosing White Coat Hypertension in Older Adults. Curr. Hypertens. Rep..

[8] Chen Y., Zhang D.Y., Li Y., Wang J.G. (2018). The Role of Out-of-Clinic Blood Pressure Measurements in Preventing Hypertension. Curr. Hypertens. Rep..

[9] Ventola C.L. (2014). Mobile devices and apps for health care professionals: Uses and benefits. Pharm. Ther..

[10] Holopainen A. (2015). Mobile technology and health applications, what are they?. Duodecim.

[11] Joosten A., Boudart C., Vincent J.L., Vanden Eynden F., Barvais L., Van Obbergh L., Rinehart J., Desebbe O. (2019). Ability of a New Smartphone Pulse Pressure Variation and Cardiac Output Application to Predict Fluid Responsiveness in Patients Undergoing Cardiac Surgery. Anesth. Analg..

[12] Joosten A., Jacobs A., Desebbe O., Vincent J.L., Sarah S., Rinehart J., Van Obbergh L., Hapfelmeier A., Saugel B. (2019). Monitoring of pulse pressure variation using a new smartphone application (Capstesia) versus stroke volume variation using an uncalibrated pulse wave analysis monitor: A clinical decision making study during major abdominal surgery. J. Clin. Monit. Comput..

[13] Pluta M.P., Dziech M., Zachura M.N., Szczepańska A.J., Czempik P.F., Liberski P.S., Krzych Ł.J. (2022). Hemodynamic Monitoring by Smartphone-Preliminary Report from a Comparative Prospective Observational Study. J. Pers. Med..

[14] Bacariza J., Gonzalez F.A., Varudo R., Leote J., Martins C., Fernandes A., Michard F. (2023). Smartphone-based automatic assessment of left ventricular ejection fraction with a silicon chip ultrasound probe: A prospective comparison study in critically ill patients. Br. J. Anaesth..

[15] Bastawrous A., Armstrong M.J. (2013). Mobile health use in low- and high-income countries: An overview of the peer-reviewed literature. J. R. Soc. Med..

[16] McCool J., Dobson R., Whittaker R., Paton C. (2022). Mobile Health (mHealth) in Low- and Middle-Income Countries. Annu. Rev. Public Health.

[17] Dol J., Richardson B., Tomblin Murphy G., Aston M., McMillan D., Campbell-Yeo M. (2019). Impact of mobile health (mHealth) interventions during the perinatal period for mothers in low- and middle-income countries: A systematic review. JBI Database Syst. Rev. Implement. Rep..

[18] Ogedegbe G., Pickering T. (2010). Principles and techniques of blood pressure measurement. Cardiol. Clin..

[19] Sharman J.E., Tan I., Stergiou G.S., Lombardi C., Saladini F., Butlin M., Padwal R., Asayama K., Avolio A., Brady T.M. (2023). Automated ‘oscillometric’ blood pressure measuring devices: How they work and what they measure. J. Hum. Hypertens..

[20] Alpert B.S., Quinn D., Gallick D. (2014). Oscillometric blood pressure: A review for clinicians. J. Am. Soc. Hypertens..

[21] Matsumura K., Rolfe P., Toda S., Yamakoshi T. (2018). Cuffless blood pressure estimation using only a smartphone. Sci. Rep..

[22] Frey L., Menon C., Elgendi M. (2022). Blood pressure measurement using only a smartphone. NPJ Digit. Med..

[23] Bard D.M., Joseph J.I., van Helmond N. (2019). Cuff-Less Methods for Blood Pressure Telemonitoring. Front. Cardiovasc. Med..

[24] Boulos M.N., Wheeler S., Tavares C., Jones R. (2011). How smartphones are changing the face of mobile and participatory healthcare: An overview, with example from eCAALYX. Biomed. Eng. Online.

[25] Caillat M., Degott J., Wuerzner A., Proençain M., Bonnier G., Knebel J.F., Stoll C., Christen U., Durgnat V., Hofmann G. (2022). Accuracy of blood pressure measurement across BMI categories using the OptiBP™ mobile application. Blood Press..

[26] Degiorgis Y., Proença M., Ghamri Y., Hofmann G., Lemay M., Schoettker P. (2022). Photoplethysmography-Based Blood Pressure Monitoring Could Improve Patient Outcome during Anesthesia Induction. J. Pers. Med..

[27] Desebbe O., Anas C., Alexander B., Kouz K., Knebel J.F., Schoettker P., Creteur J., Vincent J.L., Joosten A. (2022). Evaluation of a novel optical smartphone blood pressure application: A method comparison study against invasive arterial blood pressure monitoring in intensive care unit patients. BMC Anesthesiol..

[28] Hofmann G., Proença M., Degott J., Bonnier G., Lemkaddem A., Lemay M., Schorer R., Christen U., Knebel J.F., Schoettker P. (2023). A novel smartphone app for blood pressure measurement: A proof-of-concept study against an arterial catheter. J. Clin. Monit. Comput..

[29] Desebbe O., El Hilali M., Kouz K., Alexander B., Karam L., Chirnoaga D., Knebel J.F., Degott J., Schoettker P., Michard F. (2022). Evaluation of a new smartphone optical blood pressure application (OptiBP™) in the post-anesthesia care unit: A method comparison study against the non-invasive automatic oscillometric brachial cuff as the reference method. J. Clin. Monit. Comput..

[30] Desebbe O., Tighenifi A., Jacobs A., Toubal L., Zekhini Y., Chirnoaga D., Collange V., Alexander B., Knebel J.F., Schoettker P. (2022). Evaluation of a novel mobile phone application for blood pressure monitoring: A proof of concept study. J. Clin. Monit. Comput..

[31] Degott J., Ghajarzadeh-Wurzner A., Hofmann G., Proença M., Bonnier G., Lemkaddem A., Lemay M., Christen U., Knebel J.F., Durgnat V. (2021). Smartphone based blood pressure measurement: Accuracy of the OptiBP mobile application according to the AAMI/ESH/ISO universal validation protocol. Blood Press. Monit..

[32] Schoettker P., Degott J., Hofmann G., Proença M., Bonnier G., Lemkaddem A., Lemay M., Schorer R., Christen U., Knebel J.F. (2020). Blood pressure measurements with the OptiBP smartphone app validated against reference auscultatory measurements. Sci. Rep..

[33] Michard F. (2021). Hemodynamic Monitoring: Would a Pulse Oximeter Do the Job?. Crit. Care Med..

[34] Michard F., Shelley K., L’Her E. (2021). COVID-19: Pulse oximeters in the spotlight. J. Clin. Monit. Comput..

[35] Demiris G., Afrin L.B., Speedie S., Courtney K.L., Sondhi M., Vimarlund V., Lovis C., Goossen W., Lynch C. (2008). Patient-centered applications: Use of information technology to promote disease management and wellness. A white paper by the AMIA knowledge in motion working group. J. Am. Med. Inform. Assoc..

[36] https://www.iso.org/obp/ui/en/#iso:std:iso:81060:-2:ed-3:v1:en.

[37] Saugel B., Grothe O., Nicklas J.Y. (2018). Error Grid Analysis for Arterial Pressure Method Comparison Studies. Anesth. Analg..

[38] Grothe O., Kaplan A., Kouz K., Saugel B. (2020). Computer Program for Error Grid Analysis in Arterial Blood Pressure Method Comparison Studies. Anesth. Analg..

[39] Juri T., Suehiro K., Kanematsu R., Takahashi K., Fujimoto Y., Tanaka K., Mori T. (2022). Validation of Continuous Noninvasive Blood Pressure Monitoring Using Error Grid Analysis. Anesth. Analg..

[40] Juri T., Suehiro K., Uchimoto A., Go H., Fujimoto Y., Mori T., Nishikawa K. (2021). Error grid analysis for risk management in the difference between invasive and noninvasive blood pressure measurements. J. Anesth..

[41] Kho E., van der Ster B.J.P., van der Ven W.H., Vlaar A.P.J., Immink R.V., Veelo D.P. (2022). Clinical agreement of a novel algorithm to estimate radial artery blood pressure from the non-invasive finger blood pressure. J. Clin. Anesth..

[42] Eley V., Christensen R., Guy L., Wyssusek K., Pelecanos A., Dodd B., Stowasser M., van Zundert A. (2021). ClearSight™ finger cuff versus invasive arterial pressure measurement in patients with body mass index above 45 kg/m^2^. BMC Anesthesiol..

[43] Festo C., Vannevel V., Ali H., Tamrat T., Mollel G.J., Hlongwane T., Fahmida K.A., Alland K., Barreix M., Mehrtash H. (2023). Accuracy of a smartphone application for blood pressure estimation in Bangladesh, South Africa, and Tanzania. NPJ Digit. Med..

[44] Michard F., Barrachina B., Schoettker P. (2019). Is your smartphone the future of physiologic monitoring?. Intensive Care Med..

[45] Michard F. (2021). Toward Smart Monitoring with Phones, Watches, and Wearable Sensors. Anesthesiol. Clin..

[46] Michard F. (2017). Smartphones and e-tablets in perioperative medicine. Korean J. Anesthesiol..

[47] Michard F., Gan T.J., Kehlet H. (2017). Digital innovations and emerging technologies for enhanced recovery programmes. Br. J. Anaesth..

[48] Michard F. (2017). A sneak peek into digital innovations and wearable sensors for cardiac monitoring. J. Clin. Monit. Comput..

[49] O’Loughlin K., Neary M., Adkins E.C., Schueller S.M. (2019). Reviewing the data security and privacy policies of mobile apps for depression. Internet Interv..

[50] Xiang D., Cai W. (2021). Privacy Protection and Secondary Use of Health Data: Strategies and Methods. Biomed. Res. Int..

[51] Huckvale K., Torous J., Larsen M.E. (2019). Assessment of the Data Sharing and Privacy Practices of Smartphone Apps for Depression and Smoking Cessation. JAMA Netw. Open.

